# Detailed Shoulder MRI Findings in Manual Wheelchair Users with Shoulder Pain

**DOI:** 10.1155/2014/769649

**Published:** 2014-08-11

**Authors:** Melissa M. B. Morrow, Meegan G. Van Straaten, Naveen S. Murthy, Jonathan P. Braman, Elia Zanella, Kristin D. Zhao

**Affiliations:** ^1^Division of Orthopedic Research, Department of Orthopedic Surgery, Mayo Clinic, Rochester, MN 55905, USA; ^2^Division of Musculoskeletal Radiology, Department of Radiology, Mayo Clinic, Rochester, MN 55905, USA; ^3^Department of Orthopedic Surgery, University of Minnesota, Minneapolis, MN 55455, USA; ^4^Department of Biomedical Engineering, University of Minnesota, Minneapolis, MN 55455, USA; ^5^Rehabilitation Medicine Research Center, Department of Physical Medicine and Rehabilitation, Mayo Clinic, Rochester, MN 55905, USA

## Abstract

Shoulder pain and pathology are common in manual wheelchair (MWC) users with paraplegia, and the biomechanical mechanism of injury is largely unknown. Establishing patterns of MRI characteristics in MWC users would help advance understanding of the mechanical etiology of rotator cuff disease, thus improving the logic for prescribed interventions. The purpose of this study was to report detailed shoulder MRI findings in a sample of 10 MWC users with anterolateral shoulder pain. The imaging assessments were performed using our standardized MRI Assessment of the Shoulder (MAS) guide. The tendon most commonly torn was the supraspinatus at the insertion site in the anterior portion in either the intrasubstance or articular region. Additionally, widespread tendinopathy, CA ligament thickening, subacromial bursitis, labral tears, and AC joint degenerative arthrosis and edema were common. Further reporting of detailed shoulder imaging findings is needed to confirm patterns of tears in MWC users regarding probable tendon tear zone, region, and portion. This investigation was a small sample observational study and did not yield data that can define patterns of pathology. However, synthesis of detailed findings from multiple studies could define patterns of pathological MRI findings allowing for associations of imaging findings to risk factors including specific activities.

## 1. Introduction

Shoulder pain in manual wheelchair (MWC) users with paraplegia is common. The prevalence is reported in the literature to range from 30% to 75%, and shoulder pain is associated with increasing years of WC use [1–3]. Further, 63% of long-term MWC users will have a rotator cuff tear compared to 15% of a matched able-bodied group [4]. The exact biomechanical mechanism and its contribution to the increased risk of shoulder pain development in this upper extremity dependent population are largely unknown. It is theorized that mechanical impingement is responsible for the reported rotator cuff disease seen with imaging and concomitant pain [5, 6]. However, current concepts in rotator cuff disease are evolving. Subtypes of impingement have been identified including subacromial or external impingement, internal impingement, and subcoracoid impingement, each with a different mechanical etiology and treatment implication [7]. Establishing patterns of magnetic resonance imaging (MRI) characteristics in different patient populations would help advance understanding of the mechanical etiology of rotator cuff disease, thus improving the logic for prescribed interventions.

Previous investigations of MRI findings in MWC users have revealed a high prevalence of rotator cuff tears, coracoacromial (CA) ligament thickening and edema, and acromioclavicular (AC) degenerative joint disease (DJD) [4, 8–10]. Common to all previous studies is the reporting of the presence of tears and pathology in the AC joint and CA ligament; however, results were not expanded to include the specific location of the tear or degree of tendinopathy present. Further, the morphology of the acromion and pathology related to the bursa, labrum, or glenohumeral (GH) joints have not been described in MWC users.

Defining patterns of shoulder pathology on MRI in the MWC user population has not been possible thus far due to the paucity of studies and the limited reporting of findings. Further, the sample sizes are often small and have heterogeneity of confounding factors such as exposure to overhead sports. In order to accommodate for small heterogeneous sample sizes, consideration should be given to expanding imaging findings that are reported by including details such as tear size and location, severity of tendinopathy, joint degeneration, and condition of the labrum whenever MRI findings are reported. The synthesis of detailed imaging findings from multiple sources along with the reporting of additional risk factors will aid in the establishment of patterns of MRI characteristics in the MWC user population. The purpose of this study was to report detailed shoulder MRI findings in a sample of 10 MWC users with anterolateral shoulder pain that were participating in a shoulder pain rehabilitation intervention.

## 2. Methods

### 2.1. Study Participants

Participants who use manual wheelchairs (*n* = 10, 9 males, 1 female) were recruited as a sample of convenience from area clinics and organizations that provide treatment and services for individuals with disabilities. Participants in the current study were originally recruited for two larger studies investigating (1) shoulder rehabilitation and (2) daily exposure to shoulder elevation and upper extremity loading. A subsample of participants was included in the current MRI study based on participant availability and willingness to undergo an MRI. As such, there was no a priori power calculation performed for the MRI substudy. The Mayo Clinic Institutional Review Board approved the study protocol and informed consent was obtained from all participants before initiating test procedures.

Participants were eligible for the study if they were between 18 and 65 years of age, had a spinal cord injury, used a manual wheelchair as their primary means of mobility for a minimum of one year, were able to perform transfers and sit independently, and had shoulder pain with a date of onset no sooner than 2 weeks from the date of consent. Participants were excluded if they had cognitive impairments that limited their ability to independently follow instructions, if they had significant traumatic injury to the shoulder in which preinjury status was not attained, or if they were unwilling to have an MRI of one shoulder. The aforementioned criteria were screened over the phone. A licensed physical therapist (Meegan G. Van Straaten) performed a physical examination for additional screening. If shoulder pain was deemed to be of nonshoulder etiology (i.e., cervical spine), or if there was a presence of adhesive capsulitis (loss of greater than 25% of range of motion) or gross instability, participants were excluded from the study. Participants were required to have pain as indicated by one of the following: positive Neer, Hawkins-Kennedy or Empty Can sign, pain with humeral elevation, or complaints of anterior lateral shoulder pain during activity [11–13]. Self-reported shoulder pain was measured with the Wheelchair User's Shoulder Pain Index (WUSPI) [14]. Prior to the MRI, patients were required to meet Mayo Clinic requirements for MRI compatibility including specific screening for implanted metal within the body. Detailed demographic data was recorded including information on job type and recreational activities for the purposes of identifying participation in overhead work or competitive sports.

### 2.2. Magnetic Resonance Imaging of the Shoulder

The most painful shoulder was imaged using a standard clinical noncontrast imaging protocol. All participants were imaged on a GE (Milwaukee, WI) Signa HD MRI scanner with magnet field strength of 3 Tesla using a dedicated, commercially available, shoulder coil. The participants were positioned supine with the arm and wrist in a neutral position by the participant's side. Axial, oblique sagittal, and oblique coronal proton density (TR 3200–3500; TE 32-33) and T2 FSE FS (TR 4000; TE 50) image sequences were obtained with a matrix acquisition range of 256–384 × 256, 2 NEX, and 14 cm FOV.

### 2.3. Assessment of MR Images

The images were assessed by a board-certified, fellowship-trained, musculoskeletal radiologist (Naveen S. Murthy) using a standardized MRI Assessment of the Shoulder (MAS) guide developed by the study authors (see Supplementary Material available online at http://dx.doi.org/10.1155/2014/769649). The reliability, validity, and sensitivity of the MAS guide were not assessed in this study. The radiologist was blinded to the identity and symptoms of the participants throughout the study. Tears of the supraspinatus, infraspinatus, subscapularis, and teres minor muscles were classified as partial, full, or complete, located in one of three anatomical zones: insertion, tendon, or critical zone (myotendinous junction). The region of the tear was classified as intrasubstance, bursal, or articular. For the supraspinatus, infraspinatus, and teres minor, the portion of the tendon was defined as anterior, middle, or posterior. The subscapularis portions were defined as superior, middle, or inferior. A long head of the biceps tear was categorized as partial, split, or complete with locations defined as extra-articular, intra-articular, or bicep anchor. Tendinopathy was classified as mild, moderate, or severe [15]. AC joint degenerative arthrosis was categorized as mild, moderate, or severe, and subacromial spurs and subchondral edema/cystic changes were recorded as present (+) or absent (−). The acromion was classified according to type (1, 2, 3) and whether a lateral downslope or convex undersurface was present. CA ligament thickening was categorized as present (+) or absent (−). The presence (+) or absence (−) of bursitis and location were noted as subacromial or subcoracoid. The presence (+) or absence (−) of labral irregularities, tears, and paralabral ganglion cysts were noted. Labral tear location was designated using quadrant terminology (i.e., anterior/superior; posterior/superior; anterior/inferior; posterior/inferior). Degenerative arthrosis of the GH joint was designated as mild, moderate, or severe. The subchondral edema/cystic changes of the GH joint were noted and chondromalacia was noted as mild, moderate, or severe.

### 2.4. Descriptive Data

This investigation was an observational study. Imaging findings are presented for each participant and the number of participants with each specific finding is reported.

## 3. Results

Our participants ranged in age from 24 to 58 years (mean 38 years) with levels of spinal cord injury from C 6-7 to T12 (Table 1). One of the 10 participants was a woman, and 7 currently practiced or competed in organized sports (such as wheelchair basketball) at least once a week. The participants were MWC users for an average of 14.5 ± 9.7 years and their WUSPI pain scores were 33.1 ± 26.1 (Table 1).

There were a total of 10 rotator cuff tears (partial thickness = 6, full thickness = 1, and complete = 3), across the supraspinatus (*n* = 5), infraspinatus (*n* = 2), and subscapularis (*n* = 3). Additionally, there were four tears of the long head of the biceps tendon (complete = 1 and split = 3) (Table 2). In the supraspinatus, five of 10 participants had partial tears at the insertion in either the intrasubstance or articular regions. Three tears were in the anterior portion and one was in the middle portion. One participant had a complete insertion zone tear, and nine of 10 participants had tendinopathy (mild = 3, moderate = 4, and severe = 2). Two participants had tears in the infraspinatus tendon at the insertion zone, one partial (intrasubstance, posterior portion) and one complete. Mild and moderate infraspinatus tendinopathy was observed in nine participants (mild = 5, moderate = 4). In the subscapularis, three participants had tears: one partial tear at the insertion (intrasubstance, superior portion), one full thickness tear in the tendon (superior portion), and one complete tear at the insertion. Tendinopathy in the subscapularis was present in all 10 participants (mild = 4, moderate = 5, and severe = 1). In the long head of the biceps, three of the 10 participants had intra-articular split tears and one participant had a complete tear at the insertion. Nine participants had tendinopathy in the long head of the biceps (mild = 4, moderate = 4, and severe = 1).

All participants had AC joint degenerative arthrosis (mild = 4, moderate = 4, severe = 2) (Table 3). Five participants had AC joint spurs and 8 out of 10 participants had AC joint edema. Five participants had a type 1 acromion and four participants had a type 2 acromion. Five out of 10 participants had a downsloped acromion and one participant had a convex undersurface. CA ligament thickening was present in seven of 10 participants. Subacromial bursitis was observed in nine of the 10 participants. Three participants had labral irregularities and six had labral tears, predominantly in the posterior/superior and posterior/inferior quadrants. Paralabral ganglion cysts were present in four participants. Four participants had degenerative arthrosis of the GH joint (mild = 3, moderate = 1) and chondromalacia (mild = 3, moderate = 1). One participant had GH joint edema.

## 4. Discussion

The purpose of this study was to document expanded MRI findings of shoulder pathology in MWC users with anterior-lateral shoulder symptoms. Noncontrast clinical shoulder MR images were performed on a sample of 10 MWC users with SCI (average years of MWC use 14 years). The MRI scans demonstrated tendon tears in 70% of examined shoulders, and 90% of the rotator cuff tears occurred at the tendon insertion site. The condition of the long head of the biceps and the labrum, the presence and severity of tendinopathy, and the presence and location of bursitis were among the additional factors reported. Although this sample was not large enough to define MRI characteristics specific to MWC users, we have provided the MAS guide for other investigators to follow in evaluating and reporting detailed MRI findings for the purpose of data synthesis.

Our findings support the high prevalence of shoulder CA ligament thickening, AC joint edema, and AC joint degenerative arthrosis (Table 3) that has been previously reported in MWC users with SCI [8–10]. Additionally, 50% of our participants had tears of the supraspinatus tendon at its insertion, 60% had tears in at least one of the rotator cuff tendons, and 70% had tears when including the long head of the biceps tendon. These findings support the observation that 63% of MWC users after three decades of use will have rotator cuff tears predominantly in the supraspinatus [4]. Our study is the first to report detailed location of tears and findings of widespread tendinopathy ranging from mild to severe in this population. Additionally, subacromial bursitis and labral tears were common and have not been previously reported.

Our finding that supraspinatus tears were more common than tears in the infraspinatus, subscapularis or long head of the biceps tendon was similar to shoulder imaging findings from able-bodied adult and pediatric populations [16, 17]. This is interesting as it implies that while wheelchair use increases the frequency of rotator cuff symptoms and tearing, it may not be a different location or tear type from nonwheelchair users. Studies on able-bodied populations report more articular-side partial-thickness tears than bursal or interstitial tears [16]. Articular side tears are reported to be 2-3 times more common than bursal-sided tears, and intrasubstance tears are the least common [17, 18]. Our small sample differed in that we found more intrasubstance tears than articular-side tears (4 compared with 2) and no bursal-side tears were observed. The majority of our partial-thickness tears were at the insertion which is similar to the findings reported from a recent study of 201 able-bodied pediatric participants [16]. Tendon tears at the insertion site have been attributed to younger populations, and in the nonwheelchair adult population, the critical zone has been found to be the most common site of tears [19–21]. Our sample had an average age at injury of 24 years which may provide some explanation for the presence of insertion tears that are more common in younger and pediatric populations.

Eight of our 10 subjects are currently or were previously exposed to overhead sports or occupations. Of those seven, five had labral tears which is associated with overhead sports. Tears of the posterior supraspinatus or anterior infraspinatus, as a result of internal impingement during the cocking phase of a throw, have been documented in overhead athletes [22]. Two of our subjects who were exposed to overhead sports or occupations had a partial tear of the supraspinatus, both in the anterior portion of the tendon. More data is needed to determine whether shoulder pathology in overhead wheelchair athletes differs from overhead able bodied athletes, and whether shoulder pathology in overhead wheelchair athletes differs from MWC users who do not participate in overhead sports.

Mechanistic interpretation of these findings is limited due to the small sample size and heterogeneity with regard to factors such as age at first wheelchair use, years in chair, and exposure to overhead activity. Our sample was comprised of individuals who participate or have participated in sports, some of which are overhead. Without longitudinal imaging or comparison groups of more sedentary MWC users, we cannot conclude that the pathological findings are related to specific job or recreational activities. While the participants are described as having shoulder pain, two of the 10 have WUSPI scores below five. Since it has been documented that asymptomatic individuals can have positive MRI findings, it would have been beneficial to compare MRI results between participants' painful and nonpainful shoulders. One musculoskeletal radiologist reviewed all the images, and although he was masked to the symptoms and participant characteristics, the study would be more robust with multiple radiological reviewers. At the same time, however, this type of single-radiologist review is common in the clinical setting and is the most frequent way that imaging is used to diagnose patients. Cross-sectional studies such as ours cannot determine cause or etiology; therefore, longitudinal data is needed to truly advance the understanding of pathology development in this population. Future investigations should determine the reliability and validity of the MAS guide.

## 5. Conclusion

In our detailed reporting of shoulder MRI assessment on 10 MWC users with spinal cord injury, we observed that the tendon most commonly torn was the supraspinatus at the insertion site in the anterior portion in either the intrasubstance or articular region. Additionally, widespread tendinopathy, CA ligament thickening, subacromial bursitis, labral tears, and AC joint degenerative arthrosis and edema were common in our sample. Further reporting of detailed shoulder imaging findings is needed to confirm patterns of tears in MWC users regarding probable tendon tear zone, region, and portion. This investigation was a small sample observational study and did not yield data that can define patterns of pathology. However, synthesis of detailed findings from multiple studies could define patterns of pathological MRI findings allowing for associations of imaging findings to risk factors including activities.

## Supplementary Material

Standardized MRI Assessment of the Shoulder (MAS) guide developed by study authors.

## Figures and Tables

**Table 1:** Participant demographics, athletic involvement, and WUSPI scores.

Subject	Age	Injury level	Years of WC use	Current athletics	Previous athletics	Overhead occupation	WUSPI	Imaged side and dominancy
1	26	T9	9.4	Overhead sports, throwing			78.9	R, D
2	25	T12	21.5	Overhead sports, throwing, weight-lifting			62	R, D
3	48	T4	16.1	Endurance, ski/skate			4.9	R, D
4	45	T12	3.7	Outdoorsman, endurance, ski/skate		Yes—high force	45.1	R, D
5	32	C 6/7	6.5	Overhead sports			21.7	L, ND
6	35	T12	33.2	Overhead sports			1.2	R, D
7	33	T10	18.8	Endurance, ski/skate, overhead sports			23.9	R, D
8	32	T5	8.9	Outdoorsman, weight-lifting	Contact sport		19	R, ND
9	57	T10	3.6	Outdoorsman	Contact sport	Yes—high force	17.1	R, D
10	59	T12	23.1	Overhead sports, endurance, outdoorsman		Yes—low force	57.4	R, D

WUSPI = Wheelchair User's Pain Index.

R = right, L = left, D = dominant, ND = nondominant.

**Table 2:** Detailed MRI findings of the rotator cuff and long head of the biceps tendons.

Subject	Supraspinatus			Infraspinatus			Subscapularis			Biceps		
	Tear	Location	Tendop	Tear	Location	Tendop	Tear	Location	Tendop	Tear	Location	Tendop
1			Mod			Mild			Mod			Mod
2			Mild			Mild			Mod			Mild
3			Mod			Mild			Mod			Mod
4	P	Insert, Artic, Ant	Severe			Mod			Mild			Severe
5			Mild	P	Insert, Intra, Ant	Mod			mild			Mild
6			Mild			Mod			Mild	S	Intra	Mod
7	P	Insert, Artic, Ant	Mod			Mild			Mild			Mild
8	P	Insert, Intra, Ant	Mod			Mild	P	Insert, Intra, Sup	Mod	S	Intra	Mild
9	P	Insert, Intra, Ant	Severe			Mod	F	Tendon, Sup	Mod	S	Intra, Anchor	Mod
10	C	Insert		C	Insert		C	Insert	Severe	C	Insert	

Tears of the supraspinatus, infraspinatus, subscapularis, and teres minor muscles were classified as partial (P), full (F), or complete (C) located in one of three anatomical zones: insertion (Insert), tendon, or critical (Crit) zone. The region of the tear was classified as intrasubstance (Intra), bursal (Burs), or articular (Artic). For the supraspinatus, infraspinatus, and teres minor, the portion of the tendon was defined as anterior (Ant), middle (Mid), or posterior (Post). The subscapularis portions were defined as superior (Sup), middle, or inferior. A long head of the biceps tear was categorized as partial, split (S), or complete (C) with locations defined as extra-articular, intra-articular (Intra), or bicep anchor (Anchor). Tendinopathy was classified as mild, moderate (Mod), or severe.

**Table 3:** Detailed MRI findings of the acromioclavicular (AC) joint, acromion, coracoacromial (CA) ligament, bursa, labrum, and glenohumeral (GH) joint.

Subject	AC Joint			Acromion			CA Ligament	Bursitis		Labrum				GH Joint		
	Arthros	Spurs	Edema	Type	Downslope	Convex	Thickening	Yes/No	Location	Irreg	Tear	Location	Ganglion	Degen Arthrosis	Edema	Chond
1	Mod	−	+	2	−	−	−	+	Subacr	−	+	Postsup	+		−	
2	Mild	−	−	1	+	−	+	+	Subacr	−	−		−		−	
3	Mod	+	+	1	+	−	+	+	Subacr	−	−		−	Mild	−	Mild
4	Severe	+	+	1	+	+	+	+	Subacr	+	+	Postsup	−	Mild	−	Mild
5	Mild	−	+	1	+	−	+	+	Subacr	−	−		−		−	
6	Mild	−	−	1	−	−	−	+	Subacr	−	+	Postinf	+		−	
7	Mod	+	+	1	+	−	+	+	Subacr	−	+	Antsup, Postsup	−		−	
8	Mild	−	+	2	−	−	−	+	Subacr	−	−		−		−	
9	Mod	+	+	2	−	−	+	+	Subacr	+	+	Postsup	+	Mod	+	Mod
10	Severe	+	+	2	−	−	+	−		+	+	Postinf	+	Mild	−	Mild

AC joint degenerative arthrosis was categorized as mild, moderate (Mod), or severe, and subacromial spurs and subchondral edema/cystic changes were recorded as present (+) or absent (−). The acromion was classified according to type (1, 2, 3) and whether a lateral downslope or convex undersurface was present (+). CA ligament thickening was categorized as present (+) or absent (−). The presence (+) or absence (−) of bursitis and location was noted as subacromial or acronym (subacr) subcoracoid. The presence (+) or absence (−) of labral irregularities, tears, and paralabral ganglion cysts was  noted. Labral tear location was designated with anterior/superior (Antsup), posterior/superior (Postsup), anterior/inferior, or posterior/inferior (Postinf). Degenerative arthrosis of the GH joint was designated as mild, moderate (Mod), or severe. The presence (+) or absence (−) of subchondral edema/cystic changes was  noted and chondromalacia was noted as mild, moderate (Mod), or severe.
