# Supplementary material for: Detailed Shoulder MRI Findings in Manual Wheelchair Users with Shoulder Pain
Source: Biomed Res Int. 2014 Aug 11;2014:769649. doi: 10.1155/2014/769649 (PMC4142383; doi:10.1155/2014/769649)
Supplement: Supplementary file 1 — Standardized MRI Assessment of the Shoulder (MAS) guide developed by study authors. [file 769649.f1.pdf]

MRI Assessment of the Shoulder

Supraspinatus

Tear(s) No/Partial/Full/Complete
Location(s) Insertion/tendon/critical zone
Intrasubstance/bursal/articular
ant/mid/post

Tendinopathy 0/mild/moderate/severe
Location(s) ant/mid/post

Atrophy 0/mild/moderate/severe

Infraspinatus

Tear(s) No/Partial/Full/Complete
Location(s) Insertion/tendon/critical zone
Intrasubstance/bursal/articular
ant/mid/post

Tendinopathy 0/mild/moderate/severe
Location(s) ant/mid/post

Atrophy 0/mild/moderate/severe

Teres minor

Tear(s) No/Partial/Full/Complete
Location(s) Insertion/tendon/critical zone
Intrasubstance/bursal/articular
ant/mid/post

Tendinopathy 0/mild/moderate/severe
Location(s) ant/mid/post

Atrophy 0/mild/moderate/severe

Subscapularis

Tear(s) No/Partial/Full/Complete
Location(s) Insertion/tendon/critical zone
Intrasubstance/bursal/articular
sup/mid/inf

Tendinopathy 0/mild/moderate/severe
Location(s) sup/mid/inf

Atrophy 0/mild/moderate/severe

Biceps

Tear(s) No/Partial/Split/Complete
Location(s) Extra-articular/Intra-articular/Bicep anchor

Tendinopathy 0/mild/moderate/severe
Location(s) Extra-articular/Intra-articular

AC joint

Degen arthrosis 0/mild/moderate/severe
Subacromial spurs? YES/NO
Subchondral edema/cystic change? YES/NO

Acromion

Type **I/II/III**
Lateral downsloping YES/NO
Convex undersurface YES/NO

CA Ligament

Thickening? YES/NO

Bursitis

YES/NO
Location(s) Subacrom/Subcorac

Labrum

Degenerative Irregularity? YES/NO

SLAP Tears? YES/NO
Location(s) AntSup/PostSup/AntInf/PostInf

Paralabral Ganglions? YES/NO

Glenohumeral joint

Degen arthrosis 0/mild/moderate/severe
Subchondral edema/cystic change? YES/NO
Chondromalacia 0/mild/moderate/severe

Other
